# Risk of priapism after dynamic penile Doppler ultrasound: Single‐centre experience on a large cohort of patients

**DOI:** 10.1111/andr.70063

**Published:** 2025-05-14

**Authors:** Giuseppe Grande, Andrea Graziani, Raffaele Scafa, Andrea Delbarba, Nicola Caretta, Pierfrancesco Palego, Alberto Ferlin

**Affiliations:** ^1^ Department of Systems Medicine Unit of Andrology and Reproductive Medicine University Hospital of Padova Padua Italy; ^2^ Department of Medicine University of Padova Padua Italy; ^3^ Department of Clinical and Experimental Sciences SSD of Endocrinology ASST Spedali Civili of Brescia Brescia Italy

**Keywords:** alprostadil, dynamic penile doppler ultrasound, erectile dysfunction, *induratio penis plastica*, Priapism

## Abstract

**Objectives:**

To evaluate the risk of priapism in patients undergoing Dynamic—Penile duplex ultrasound (D‐PDU) who were referred to our Unit between January 2022 and December 2023.

**Patients and methods:**

We enrolled 292 patients, of whom 268 underwent Dynamic—Penile duplex ultrasound for erectile dysfunction and 24 for Peyronie's disease. The mean age of the patients was 56 ± 12 years, the mean alprostadil dose administered was 9.17 ± 5.59 mcg and the mean erection response was 79.55 ± 19.95%. To evaluate the occurrence of priapism, we considered i) patients who called our phone number within the first hours following the exam; ii) patients who were referred to our Emergency Department within the 24 h following Dynamic—Penile duplex ultrasound; iii) patients who reported to us the occurrence of priapism at subsequent follow‐up visit; iv) patients who e‐mailed us to report this side effect.

**Results:**

We found no cases of priapism (0/292 patients, 0%). Therefore, statistical analysis with correlation and regression analysis was not conducted.

**Conclusions:**

In our opinion, the risk of priapism following Dynamic—Penile duplex ultrasound with alprostadil injection might be re‐evaluated, as it appears to be a rare and preventable condition.

## 1 INTRODUCTION

Priapism is defined as a condition characterized by persistent penile erection lasting more than 4 h.^1^, ^2^ It is a rare condition—resulting in 5.3 emergency department visits per 100 000 patient‐years in the US^2^ – which requires an emergent management of this disease directed toward achieving detumescence. If this medical condition is left untreated, penile corporal tissue necrosis and fibrosis result along with permanent erectile dysfunction (ED). The causes of ischemic priapism include, among others, vasoactive medications, such as alprostadil, which have been blamed for an increased incidence of this disorder and are thought to cause at least 25% of all cases.^3^

The occurrence of priapism, especially following intracavernous alprostadil injection, is particularly elevated in patients at higher risk due to various underlying conditions. These include haematological disorders (e.g. sickle cell disease, thalassemia and leukaemia), neurological disorders (e.g. spinal cord injuries and multiple sclerosis), pelvic surgery or diseases, the use of certain medications (e.g. phosphodiesterase type 5 inhibitors [PDE5Is] and antipsychotics), and young individuals with higher risk of psychogenic ED, due to the fact that they might be more prone to overreacting to alprostadil injection.^4^, ^5^, ^6^, ^7^, ^8^ Table 1 resumes the clinical conditions associated with higher risk of priapism and the relative pathophysiological mechanisms involved.

**TABLE 1:** Clinical conditions associated with a higher risk of priapism and etiological mechanisms.

Category	Specific conditions	Aetiology
Hematological disorders	‐ Sickle cell anaemia ‐ Leukaemia ‐ Thalassemia ‐ Multiple myeloma	Increased blood viscosity and obstruction of venous outflow
Neurological disorders	‐ Spinal cord injury ‐ Cauda equina compression ‐ Multiple sclerosis ‐ Autonomic neuropathy ‐ Cerebrovascular accident	Disruption of neural pathways regulating penile detumescence
Substance abuse	‐ Alcohol abuse ‐ Recreational drugs (e.g. cocaine)	Vascular effects or neurotransmitter disruptions
Pharmacological treatments	‐ Alpha‐adrenergic receptor antagonists ‐ Antidepressants ‐ Antipsychotics ‐ Anticoagulants ‐ Phosphodiesterase inhibitors	Vasodilation or neurotransmitter imbalance
Malignancies	‐ Penile or pelvic tumours	Obstruction of venous drainage or paraneoplastic syndromes
Post‐surgical	‐ Prostate surgery	Alteration of normal neuro‐vascular pathways
Trauma	‐ Penile or pelvic trauma	Damage to venous drainage pathways
Physiological factors	‐ Young age	Overreaction to pharmacological stimulation

Whilst priapism during less than 24 h might be managed non‐surgically in most cases (being managed, for instance, with corporal blood aspiration followed by instillation of phenylephrine into the corpus cavernosum), priapism which lasts more than 36 h is frequently associated with permanent ED.^9^, ^10^

Current American Urological Association Priapism Treatment Guidelines^11^ report that oral therapy is not recommended as the only treatment for ischemic priapism. Indeed, for ischemic priapism, diluted phenylephrine is the recommended agent for intracavernosal injections as it has fewer adverse cardiac effects than alternative drugs. The phenylephrine must be diluted before use. The recommended dilution is 100 to 500 mcg/mL. Injections can be performed every 5 min for up to 3 additional injections. Others have recommended repeat injections for up to 60 min or a maximum total dosage of 1000 mcg of phenylephrine.

Dynamic—Penile duplex ultrasound (D‐PDU) is a diagnostic tool mostly used to evaluate the presence, severity, or type of ED.^12^ It might also be used to evaluate the presence and the severity of post‐traumatic urethral stricture^13^ and the presence and features of Peyronie's disease (PD), also known as *induratio penis plastica*.^12^ In order to avoid risk related to D‐PDU, it has been proposed the evaluation of its most useful parameter, the peak systolic velocity (PSV), in the flaccid state, albeit this evaluation seems not to be very useful for the purpose of an accurate vascular imaging study of the erectile dynamics.^12^ Therefore, in order to study properly the presence and the severity of ED or PD, D‐PDU is currently recommended in clinical practice.

However, frequent concerns about D‐PDU regard the possible risk of priapism. The risk might appear even higher in light of the evidence of the use of D‐PDU to study and diagnose ED, a condition which is becoming more common and more studied.

In this manuscript, we evaluated the risk of priapism in patients referring to our Unit who undergo D‐PDU, further discussing the real risk of priapism following D‐PDU. The aim of the study was therefore to evaluate the incidence and risk factors of D‐PDU—induced priapism in a cohort of patients referring to our Unit.

## 2 MATERIALS AND METHODS

We performed a retrospective, single‐centre study, evaluating patients referring to our Unit who executed D‐PDU between January 2022 and December 2023.

D‐PDU is performed with the supine‐lying patient, starting with a grayscale baseline study of penile shape and erectile tissue, in order to exclude lesions of these structures. This first phase is followed by intracavernous injection (ICI) of alprostadil. Intracavernous alprostadil (synthetic prostaglandin E1) is a vasodilating agent which acts by relaxing the smooth muscles of the corpus cavernosum and by increasing the diameter of cavernous arteries; this leads to erection. At therapeutic doses, intracavernous alprostadil is well tolerated. The most common adverse event of transient penile pain occurred in around one‐third of patients and in 11% of injections, causing 3%–5% of patients to withdraw from self‐injection programmes, whilst potentially serious adverse events such as priapism and fibrosis have been previously reported in 8% of patients.^14^ Besides morphological features, artery calibre and symmetry and anatomic variants, the most important parameters that might be evaluated during D‐PDU are the PSV, the end‐diastolic velocity (EDV), the resistive index (RI) and the acceleration time (AT). Normal parameters measurable during D‐PDU are reported in Table 2.

**TABLE 2:** Normal vascular parameters measurable during Dynamic—Penile duplex ultrasound (D‐PDU).

Parameter	Abbreviation	Measure unit	Normal values
Peak systolic velocity	PSV	cm/s	≥35 cm/s
End‐diastolic velocity	EDV	cm/s	<3–5 cm/s
Resistive index	RI	///	>0.9
Acceleration time	AT	ms	<110 ms

Before the execution of D‐PDU, we gave patients written and oral consent regarding D‐PDU, informing patients regarding the risk of priapism (defined as the presence of erection prolonged for more than 3 h after the execution of the exam).

We moreover arranged the dosage of alprostadil according to the risk factors of patients. In detail, albeit maintaining a standard dose of 10 mcg, we used a lower dose of 5 mcg in patients with one of the following criteria: young patients (<40 years old); patients with neurological disorders; patients with recent prostatic surgery (<6 months); severe liver dysfunction; haematological disorders. Furthermore, we used a dose of 2.5 mcg if 2 factors were simultaneously present. In addition, we suggested patients avoid taking PDE5Is 24 h before the exam (72 h for tadalafil). In patients receiving a reduced dose of alprostadil (below 10 mcg), such as young patients, in case the used dose was insufficient to obtain the erection after 15 min, we used a redosing protocol, administering the remaining dose of alprostadil in order to have a total quantity of 10 mcg administered to the patient.

We instructed patients, in case of persistent erection for more than one hour, to have physical activity, sexual activity or a short walk. We moreover instructed patients to call the specialist who executed the exam in case of persistent erection for more than 3 h (giving patients our work phone number and e‐mail address) and to inform our centre, in case of priapism, as soon as possible. Furthermore, we suggested patients to refer to our Emergency Room (ER) Department if needed.

In our unit, the detumescence reversal protocol for treating priapism has been developed. Patients with an erection lasting more than 3 h should receive an ICI of the sympathomimetic Etilefrine hydrochloride at a dosage of 500 mcg, with a second injection given after 5 min if necessary.

In our study, we evaluated D‐PDU parameters, age, the presence of chronic diseases and chronic therapies (focusing in particular on therapy for ED/PD). Therefore, in order to evaluate the presence of priapism, we considered i) patients who called us on our phone after the exams; ii) patients who were referred to our ER Department in the 24 h following D‐PDU; iii) patients who told us, at the following visit, the occurrence of priapism iv) patients who e‐mailed us to inform about this side effect of D‐PDU.

We, therefore, considered the categories of patients at higher risk of priapism: i) young patients (age* <* 40 years old); ii) patients with neurological disorders (clinical history of spinal cord injuries or multiple sclerosis); iii) patients with recent prostatic or pelvic surgery in the last 12 months; iv) known history of severe liver dysfunction (known severe cirrhosis or elevated transaminases more than 3x upper limits of the normal in the last 6 months); v) haematological disorders (such as sickle cell disease, thalassemia and leukaemia); iv) use of medication which are risk factors for priapism (such as PDE5Is or antipsychotics) (although, as previously exposed, we always suggest patients to avoid taking PDE5Is 24 h before the exam – 72 h for chronic tadalafil therapy).

There is no universal definition of ‘young subject’ across the literature. However, previous studies on drug‐induced priapism^15^ have shown an increased risk of priapism in patients in the 4th decade of age, compared to those in their 50s. Based on this evidence, our group selected 40 years as the cut‐off, acknowledging that this threshold is relative rather than absolute. It is also plausible that the risk may be even higher in younger individuals.

## 3 RESULTS

We enrolled 292 patients, referring to our Unit between January 2022 and December 2023. Patients’ data are summarized in Table 3.

**TABLE 3:** General data of patients considered.

Parameter evaluated in 292 patients	Results (mean ± SD)
Mean age (years)	56.10 ± 12.15
Number of patients executing the exam due to ED	268/292 (91.78%)
Number of patients executing the exam due to PD	24/292 (8.22%)
Number of patients taking chronic PDE5‐I	63/292 (21.58%)
Number of patients taking PDE5‐I as needed	41/292 (14.04%)

Abbreviations: D‐PDU, Dynamic—Penile Doppler ultrasound; ED, Erectile dysfunction; PD, Peyronie's disease; PDE5‐I, Phosphodiesterase type 5 inhibitor; SD, Standard deviation.

The average age of our patients was 56.10 ± 12.15 years. 268/292 (91.78%) patients underwent the exam due to suspected or known ED and 24/292 (8.22%) due to suspected or known PD. 63/292 (21.58%) patients were taking chronic PDE5‐I, whilst 41/292 (14.04%) were taking PDE5‐I as needed.

The mean used alprostadil dose was 9.17 ± 5.59 mcg and the mean evaluated erectile response was 79.55 ± 19.95%. Vascular parameters are summarized in Table 4.

**TABLE 4:** Technical, ultrasound and vascular Dynamic—Penile duplex ultrasound (D‐PDU) data of the patients.

Parameter evaluated in 292 patients	Results (mean ± SD)
Mean alprostadil dose (mcg)	9.17 ± 5.59
Mean evaluated response (%)	79.55 ± 19.95
Mean right PSV (cm/s)	49.73 ± 18‐52
Mean left PSV (cm/s)	50.27 ± 20.84
Mean right EDV (cm/s)	2.04 ± 7.43
Mean left EDV (cm/s)	1.40 ± 6.98
Mean right RI	0.94 ± 0.55
Mean left RI	0.91 ± 1.14
Mean right AT (ms)	91.42 ± 28.22
Mean left AT (ms)	90.25 ± 28.13

Abbreviations: AT: Acceleration time. EDV: End‐diastolic velocity. PSV: Peak systolic velocity. RI: Resistive index. SD: Standard deviation.

When dealing with the categorization of D‐PDU results, we evaluated the parameters of D‐PDU dividing them into three subcategories: normal (normal mean PSV and normal mean EDV), arterial insufficiency (mean PSV < 35 cm/s) and veno‐occlusive dysfunction (mean EDV **≥** 5 cm/s) (Table 5). We reported that 132/292 patients (45.21%) had normal parameters, 58/292 patients (19.86%) had arterial insufficiency and 102/292 patients (34.93%) had veno‐occlusive dysfunction.

**TABLE 5:** Categorization of results of Dynamic—Penile duplex ultrasound (D‐PDU) in our cohort of 292 patients.

Categorization of D‐PDU	Normal value	Results (n/total and% of patients)
Normal	Normal PSV and EDV	132/292 (45.21%)
Arterial insufficiency	Mean PSV < 35 cm/s	58/292 (19.86%)
Veno‐occlusive dysfunction	Mean EDV **≥** 5 cm/s	102/292 (34.93%)

In our cohort of patients, we found no cases (0/292, 0%) of priapism. The data were double‐checked, and the second analysis confirmed the absence of cases of priapism in the cohort of patients who were considered.

We therefore considered the categories of patients at higher risk of priapism, reporting the following results: i) young patients: 35/292; ii) patients with neurological disorders: 4/292; iii) patients with recent prostatic or pelvic surgery: 0/292; iv) severe liver dysfunction: 0/292; v) haematological disorders: 6/292; iv) use of medication which are risk factors for priapism:113/292.

This is to underline the fact that, due to a problem with our hospital informatics system, we lost data regarding the D‐PDU exams of about 50 patients who were referred to our Unit between September and December 2022. Therefore, we could not include those extra fifty patients in our cohort of study. Notwithstanding, analyzing the available data of those extra fifty patients (follow‐up visits and evaluations, e‐mails, etc.) we did not find any case of priapism.

## 4 DISCUSSION

Priapism is a condition which requires an emergent management of this disease directed toward achieving detumescence. As said above, if left untreated, penile corporal tissue necrosis and eventually fibrosis result along with permanent erectile dysfunction. D‐PDU is a diagnostic exam which is currently used to diagnose and study the presence and severity of ED, PD or the presence of post‐traumatic fistulae or post‐traumatic disorders. In our cohort, patients underwent D‐PDU execution due to the presence of known or suspected ED and known or suspected PD. The mean erective response indicated an average good erective response, further inducing the possibility of following the risk of priapism. Nevertheless, in our cohort of 292 patients, we found no case of priapism.

A recent analysis, based on the WHO global database of records of adverse drug reactions^16^ reported that that intracavernousal drugs induced priapism 25 times higher than oral PDE5I. A previous study, by Jiang et al.^17^ evaluated the effects of low‐dose phenylephrine as a prophylaxis against iatrogenic priapism and reported 44/77 having persistent rigidity (score 4–5 out of 5) 60 min after receiving alprostadil. After conducting a literature search, they reported the incidence of iatrogenic priapism ranging from 0.26% to 10.26%, with higher incidences of iatrogenic priapism with papaverine versus alprostadil injections and lower rates of priapism with the use of alprostadil, ranging from 0.26% to 0.94%. Another retrospective study^18^ evaluating patients who presented with priapism in Western Australia during the years 1985–2000, found a total of 82 episodes of priapism in 63 patients, with 62 episodes occurring after (ICI). The Authors conclude that priapism might be an uncommon, albeit important, major side effect of ICI therapy, although its incidence has fallen with the introduction of prostaglandin E1 monotherapy as the favoured drug for ICI therapy of ED. Linet et al.^19^ investigated the efficacy and safety of ICI in three separate multi‐institutional, prospective studies in 296 men with ED and found that prolonged erections occurred in 5% of the men, whilst priapism in 1% of the time. Another study, evaluating 848 men with ED, reported a prevalence of 0.9% of priapism.^20^ In addition, Giuliano et al.^21^ evaluated 144 men with ED and found just one case of priapism. Moreover, Shamloul et al.^22^ trying to assess the value of D‐PDU in the prediction of ICI‐induced ischemic priapism in 400 patients, found 19 cases of priapism and proposed the disappearance of blood flow in the cavernous artery after an hour of sustained rigid erection as predictive of priapism with 100% specificity and sensitivity.

In our experience, no cases of priapism have been reported in a population of 292 patients after following D‐PDU.

However, it is important to acknowledge some limitations of our study. First of all, since patients were not actively monitored in the hours immediately after the examination, there remains a possibility—albeit unlikely –that some cases of priapism might have occurred but went unreported. Potential reasons for this could include recall bias, as patients may misremember or fail to disclose the event during the follow‐up visits, or loss of the patients to follow‐up visits. Together, these biases could have contributed to an underestimation of the true incidence of priapism within this cohort. In addition, we found that only 45.21% of patients evaluated had normal vascular parameters (normal PSV and normal EDV). Therefore, the fear of priapism might have led to the prevention of priapism during the ultrasound evaluation, inducing an underdosing of alprostadil and thus a high rate of false positive results. Another limitation of the study is that the evaluated cohort included a relatively low prevalence of patients at higher risk of developing priapism following alprostadil injection. Consequently, these findings cannot be applied to populations with specific conditions known to increase the risk of priapism, such as haematological disorders (e.g., sickle cell anaemia or leukaemia), neurological disorders (e.g., spinal cord injuries, multiple sclerosis), pelvic surgery or diseases and the use of certain medications and drugs.^4^, ^5^, ^6^, ^7^, ^8^, ^9^, ^23^

For these specific conditions, dedicated studies should be conducted to accurately assess the incidence of priapism and develop tailored strategies to minimize associated risks. Such strategies should include conducting the test only when absolutely necessary, reducing the dosage of alprostadil to the minimum effective amount, and ensuring close patient monitoring to enable prompt intervention in the event of complications.

It might be therefore time to re‐evaluate the risk of priapism after D‐PDU, representing indeed a condition rare and preventable. In particular, the risk of priapism might be prevented using: i) a reduction in dose of alprostadil in patients with a higher risk of priapism; ii) the indication of having physical/sexual activity or a short walk, in patients with persistent erection after one or 2 h; iii) the indication of not taking any PDE5I in the 24–48 h before the D‐PDU evaluation (72 h for tadalafil).

Anyway, providing patients with a clear and comprehensive explanation of the risks associated with D‐PDU remains essential. We, therefore, recommend instructing patients to promptly seek medical attention in the event of priapism by visiting the ER department of the nearest hospital or contacting the personnel of the Unit as soon as possible.

## 5 CONCLUSION

Priapism is a condition which requires an emergent management of this disease directed toward achieving detumescence. It is our opinion that the risk of priapism after D‐PDU might be re‐evaluated, representing indeed a condition rare and preventable.

## AUTHOR CONTRIBUTIONS

Andrea Graziani, Nicola Caretta and Pierfrancesco Palego conceived and designed the analysis; Andrea Graziani and Raffaele Scafa collected the data; Andrea Graziani performed the analysis, Andrea Graziani and Giuseppe Grande wrote the paper with input from all authors; Raffaele Scafa, Nicola Caretta, Pierfrancesco Palego and Andrea Delbarba revised the manuscript critically for important intellectual content; Alberto Ferlin supervised the study and critically revised the manuscript and gave final approval of the version to be published. All authors read and approved the final version of the manuscript.

## CONFLICT OF INTEREST STATEMENT

The authors declare no conflict of interest.

## Data Availability

The data that support the findings of this study are available from the corresponding author upon reasonable request.
